# Illness management and recovery: one-year follow-up of a randomized controlled trial in Danish community mental health centers: long-term effects on clinical and personal recovery

**DOI:** 10.1186/s12888-019-2048-0

**Published:** 2019-02-11

**Authors:** Sofie Bratberg Jensen, Helle Stentoft Dalum, Lisa Korsbek, Carsten Hjorthøj, John Hagel Mikkelsen, Karin Thomsen, Kristen Kistrup, Mette Olander, Jane Lindschou, Kim T. Mueser, Merete Nordentoft, Lene Falgaard Eplov

**Affiliations:** 1 Region Zealand, University Hospital Roskilde, Roskilde, Denmark; 2 Competence Center for Rehabilitation and, Recovery, Mental Health Center Ballerup, Ballerup, Denmark; 3 Copenhagen Research Center for Mental Health - CORE, Mental Health Center Copenhagen, Copenhagen University Hospital, Copenhagen, Denmark; 4 Community Mental Health Center Frederiksberg-Vanløse, Mental Health Center Frederiksberg, Frederiksberg, Denmark; 5 Community Mental Health Center Ballerup-Egedal-Herlev, Mental Health Center Ballerup, Ballerup, Denmark; 6 Mental Health Center Frederiksberg, Frederiksberg, Denmark; 7 Roskilde Community, Roskilde, Denmark; 8 Copenhagen Trial Unit, Center for Clinical Intervention Research, Department 7812, Rigshospitalet, Copenhagen University Hospital, Copenhagen, Denmark; 9 Boston University Center for Psychiatric Rehabilitation, Boston, USA

**Keywords:** Illness management and recovery, Rehabilitation, Psychoeducation, Randomized clinical trial, One-year follow-up, Community mental health, Severe mental illness

## Abstract

**Abstract:**

Background: Illness Management and Recovery (IMR) is a curriculum-based rehabilitation program for people with severe mental illness with the short-term aim of improving illness self-management and the long-term aim of helping people achieve clinical and personal recovery.

**Method:**

Participants with schizophrenia or bipolar disorders were recruited from three community mental health centers in the Capital Region of Denmark and randomized to receive group-based IMR and treatment as usual or only the usual intervention. All outcomes were assessed at baseline, postintervention, and the one-year follow-up. Long-term outcomes were categorized according to clinical recovery (i.e., symptoms, global functioning, and hospitalization) and personal recovery (i.e., hope and personal agency). Generalized linear mixed model regression analyses were used in the intent-to-treat analysis.

**Results:**

A total of 198 participants were included. No significant differences were found between the IMR and control groups in the Global Assessment of Functioning one year after the intervention, nor were there significant differences in symptoms, number of hospital admissions, emergency room visits, or outpatient treatment.

**Conclusion:**

The present IMR trial showed no significant effect on clinical and personal recovery at the one-year follow-up. Together with the results of other IMR studies, the present study indicates that the effect of IMR on symptom severity is unclear, which raises questions regarding the impact of IMR on functioning. Additionally, IMR did not affect personal recovery. Although more research is needed, the results indicate that the development of other interventions should be considered to help people with severe mental illness achieve a better level of functioning and personal recovery.

**Trial registration:**

Trial registered at http://www.clinicaltrials.gov (NCT01361698).

## Background

**Fig. 1:**
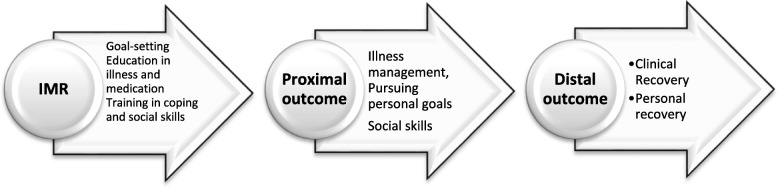
Hypothesized short-term and long-term outcome of the Illness Management and Recovery Program

The Illness Management and Recovery (IMR) program is a manualized, curriculum-based, recovery-oriented, rehabilitation program for people with schizophrenia or a major mood disorder [1]. Typically, IMR is delivered by mental healthcare professionals with an aim of helping individuals set and pursue personal recovery goals and learn illness management skills to facilitate their attainment of these goals [1]. Clinical recovery refers to a reduction in the signs and symptoms of the mental illness and restoration of cognitive, social, and occupational functioning, whereas personal recovery refers to the process of constructing a personally meaningful life within and beyond the limits of one’s mental illness [2–5]. The IMR program is based on two theoretical models: the transtheoretical model and the stress-vulnerability model. The transtheoretical model proposes that motivation to change develops over a series of stages (precontemplation, contemplation, preparation, action, maintenance) and that facilitating change requires stage-specific interventions. The stress-vulnerability model posits that the course and outcome of schizophrenia is determined by the dynamic interplay of biological vulnerability, stress, and coping. The IMR-program is aimed at interrupting the cycle of stress and vulnerability that leads to relapse and poor functioning [1, 4, 6]. Therefore, the long-term outcomes of IMR are to improve both personal and clinical recovery, as illustrated in Fig. 1.

The present study focuses on the long-term effects of the IMR program. The literature review on the IMR program by McGuire et al. (2014) [7] found two randomized clinical trials (RCTs) [8, 9] and one quasi-experimental trial [10] with results on the long-term effects of IMR. Two subsequent RCTs examined the long-term effects of IMR [11, 12], and one RCT preplanned a long-term follow-up but instead conducted a qualitative study at one year of follow-up [13]. Three of the trials revealed that the participants who received IMR had greater reductions in symptoms and increases in functioning posttreatment than those who received the usual services and that they maintained these improvements during follow-up [8, 9, 12]. However, all three trials had methodological limitations, such as not establishing a primary outcome, conducting assessments with nonblinded interviewers, and failing to perform a sample size calculation. In contrast, two RCTs by Salyers et al. (2010/2014) reported no group differences in favor of IMR at either the posttreatment or follow-up time points [10, 11]. However, these two RCTs also had methodological limitations (i.e., not including assessor-blinded interviews and primary outcomes, as noted below). The early systematic review concluded that the IMR-program was an effective intervention, but later studies have cast doubt.

Two important issues must be considered when evaluating research on the IMR-program. The first issue is exposure to IMR, which can be measured by the number of IMR sessions attended. The second issue is fidelity to the principles of the IMR program, which can be measured with the IMR Fidelity Scale [14]. One RCT that also used a modified IMR (i.e., it excluded three IMR modules) did not report the number of sessions attended or the fidelity score [15]. Another RCT reported a high degree of exposure to IMR; however, the study did not perform fidelity assessments, which limited the ability to estimate the quality of IMR implementation [9]. Levitt et al. (2009) reported high fidelity but a lower degree of exposure than Färdig et al. (2011), with 54% of participants attending more than 20 sessions [8, 9]. The two studies by Salyers et al. (2010/2014) showed a good to high fidelity score; however, the authors reported low exposure to IMR [10, 11]. Together, the five RCTs had limitations in either IMR exposure or fidelity assessment.

The present RCT was designed to improve upon the methodological limitations of previous IMR research and to evaluate whether participants in the IMR program exhibited improved clinical and personal recovery and illness management postintervention and at the one-year follow-up [16]. The IMR program was implemented with high fidelity but, unfortunately, with lower exposure to IMR than desired and reported in the study by Levitt et al. (2009), with 47% of the participants attending more than 20 sessions. No differences in any postintervention outcomes were found [17]. This article reports the one-year follow-up outcomes for clinical and personal recovery.

## Methods

### Design

This study was a prospective, assessor-blinded, RCT performed in three community mental health centers (CMHCs) in the Capital Region of Denmark, which covers a population of approximately 1.8 million people. The IMR program was provided as an add-on to treatment as usual (TAU) care in the intervention group. The trial design is described in detail elsewhere and therefore will only be described briefly here [16]. Participants were included from March 2011 to December 2013. Assessments were conducted at baseline, posttreatment, and one year following the end of treatment, with follow-up data collection conducted between December 2013 and December 2014. The present article focuses on the one-year follow-up data.

### Participants

We included participants based on diagnostic interviews with the Present State Examination [18] conducted by a psychiatrist or trained psychologist to verify the diagnosis of schizophrenia (F20.x) or bipolar disorder (F31.x) according to the ICD-10 criteria. Additional inclusion criteria were as follows: a) 18 years of age or older, b) sufficient fluency in Danish to participate in the IMR program, and c) willingness to provide informed consent. The exclusion criteria were as follows: a) having a guardian or a forensic arrangement, b) meeting the criteria for the ICD-10 diagnosis of dementia or mental retardation, c) having an active substance use disorder, d) living in a community residential home, or e) being involved in a psychoeducational course at the time of inclusion.

### Randomization and blinding

Patients were recruited from three CMHCs in the Capital Region of Denmark. Diagnostic eligibility for the study was established with the Present State Examination (PSE) administered by a trained psychiatrist or a psychologist [16, 17]. The randomization was performed centrally and by telephone by the Copenhagen Trial Unit to conceal the allocation sequence. The allocation sequence was computer-generated using permuted blocks in varying sizes of 6, 8, and 10. The level of blinding at the one-year follow-up was focused on the assessor-blinded interviews, because blinding at the data analysis level was compromised during the posttreatment investigation.

## Interventions

### The IMR program

Participants randomized to the IMR intervention group were offered an IMR course in addition to TAU as provided by CMHCs in the Capital Region of Denmark. The IMR program lasted 9 months with weekly group sessions, and each closed-enrollment group included ten patients. The group sessions were conducted by two or three clinicians at CMHCs. All patients receiving IMR were given educational hand-outs corresponding to the eleven different module topics (see Table 1).

**Table 1:** Modules in the Illness Management and Recovery Program

Module	Theme
1	Recovery strategies
2	Practical facts about mental illness
3	The Stress-Vulnerability Model
4	Building social support
5	Using medication effectively
6	Drug and alcohol use
7	Reducing relapses
8	Coping with stress and common problems
9	Coping with symptoms
10	Getting your needs met in the mental health system
11	Healthy Lifestyles

### Treatment as usual (TAU)

TAU consisted of an individually adapted interdisciplinary treatment containing medication, individual case manager support, individual and group therapy, and unstandardized psychoeducation. The staff of the CMHCs were trained mental health professionals, including nurses, psychiatrists, psychologists, physiotherapists, occupational therapists, and licensed social workers. Every patient in the CMHC had a case manager who together with the patient selected the elements of the individual’s treatment plan. The patient met with the case manager once a week (or more or less frequently depending on the state and preference of the patient) [16, 17].

### IMR Fidelity scale

The IMR fidelity assessments were performed four months after initiating the study and again at the end of each IMR group. Staff specially trained in IMR fidelity assessment from one participating community mental health centers was conducting the assessments at the other community mental health centers’ groups and vice versa. A multiple data approach was used including: interviews, observation of the IMR group, an audit of the patient’s service records as well as audit of the IMR notes of progress. Two raters independently scored each session and then discussed any discrepancies and reached a consensus rating.

## Outcomes

The assessment of clinical recovery (i.e., functioning and symptoms) was conducted through interviews with an assessor who was blinded to the treatment group allocation. Assessment of personal recovery was obtained through the participants’ self-reports. Information on illness management and recovery was obtained by the staff and was self-reported by the participants. All outcomes were assessed postintervention and again at the one-year follow-up. The primary outcome was the Global Assessment of Functioning (GAF) assessed by the GAF-F postintervention. Therefore, all one-year follow-up outcomes are secondary or explorative outcomes.

The GAF is a generic rating scale ranging from 1 (lowest) to 100 (highest) that was developed as an overall measure of the patient’s social, psychological, and occupational functioning. The GAF can be rated with a single score that reflects both the symptom severity and functional impairment or with separate scores for symptoms (GAF-S) and functioning (GAF-F) [19]. For the present study, both the GAF-F and GAF-S scores were obtained.

The Personal and Social Performance (PSP) scale measures social functioning within four domains: socially useful activities, personal and social relationships, self-care, and disturbing and aggressive behavior. The PSP provides a score between one and one hundred using a six-point severity scale for each domain. The ratings are based on the outcome of a structured clinical interview. A high score indicates higher personal and social functioning [20].

The Positive and Negative Syndrome Scale (PANSS) is a semi-structured interview pertaining to the patient’s symptoms over the past month [21, 22]. The PANSS includes 30 items with a seven-point rating that represents increasing levels of psychopathology. The PANSS includes three subscales: the Positive Scale (7 items), the Negative Scale (7 items), and the General Psychopathology Scale (16 items). The PANSS total and subscales scores are calculated by summing the ratings of all items on the total scale and each subscale (range for total: 30–210, range for Positive and Negative Scales: 7–49, and range for General Psychopathology Scale: 16–112) [22].

The 6-item Hamilton Rating Scale for Depression (HAM) is an interview-based measure of depression that includes the following six items: depressed mood, work and activities, sleep disturbance, guilt, anxiety, and retardation [23]. The HAM-D6 is interview-based and clinician-rated on a five-point scale (0–4), except for sleep disturbance, which is rated on a three-point scale (0–2). The HAM-D6 minimum score is zero, and the maximum score is 22.

The Young Mania Rating Scale (YMRS) are composed of the following 11 item scale as follows: 1. Elevated Mood, 2. Increased Motor Activity-Energy, 3. Sexual Interest, 4. Sleep, 5. Irritability, 6. Speech Rate and Amount, 7. Language Thought Disorder, 8. Content, 9. Disruptive-Aggressive Behavior, 10. Appearance, and 11. Insight [24]. The YMRS is a clinician-rated instrument that is used to assess the severity of mania; items 1, 2, 3, 4, 7, 10, and 11 use a five-point scale, and items 5, 6, 8, and 9 use a nine-point scale. The YMRS minimum score is zero, and the maximum score is sixty [25].

The IMR scale (IMRS) was developed to measure illness self-management outcomes based on the stress-vulnerability model and is an integrated part of the IMR program’s Implementation Toolkit. Each item is rated on a five-point scale, with lower scores reflecting lower levels of illness management. The IMRS can be scored by summing up all items on the scale for the total score (range: 15–75) or for three subscales based on factor analyses with a three-factor solution for the subscales (Recovery scale, Management scale, and Biology scale) [13, 26].

The Mental Health Recovery Measure (MHRM) is a self-reported 30-item scale that assesses perceived recovery for individuals with a serious mental illness [27]. The items are rated on a five-point scale ranging from zero (strongly disagree) to four (strongly agree), creating a total score range of zero to 120. Higher MHRM scores indicate higher self-reported levels of mental health recovery.

The Adult Hope Scale is a self-reported six-item scale that assesses the level of hope. The items are assessed using an eight-point Likert scale ranging from definitely false to definitely true [28]. The measure consists of two subscales: agency (goal determination) and pathways (extent of belief in ability to achieve goals). The Hope Scale minimum score is six, and the maximum score is 48.

The Client’s Satisfaction Questionnaire measures the participants’ satisfaction with community mental health treatment [29], with a score range of 8–32.

Finally, we collected data from the following national registers for all participants regarding their service utilization: the Civil Registration System (CPR-register), Danish Psychiatric Central Research Register (PCRR), Danish Register of Causes of Death, and Danish National Patient Register-Psychiatry [30]. We collected data on the number of hospital admissions, length of admissions in days, and number of emergency service visits. Every inhabitant of Denmark is given a unique ten-digital personal identification number, and therefore, complete follow-up using national databases is possible.

## Statistical analysis

A detailed statistical analysis plan was created before performing any analyses. The sample size calculation was conducted for the posttreatment investigation but not for the one-year follow-up. The analyses were conducted according to the intent-to-treat principle, with the two-tailed level of significance for all statistical tests set at 0.05. For the one-year follow-up analyses, we used generalized linear mixed-effects regression analyses. The relative changes in the GAF-F and social scores, psychiatric symptoms and illness management, and recovery were evaluated postintervention and at the one-year follow-up using the baseline data as covariates. Missing data were handled through multiple imputations, which were conducted with a linear mixed model, with post-intervention and follow-up as repeated measurements, baseline as a fixed effect covariate, and an unstructured covariance matrix. We assumed and estimated a time trend for the measures for the postintervention and one-year follow-up measurements. Furthermore, the linear mixed models were selected for their capacity to handle missing longitudinal data. The fixed values were the baseline values of GAF-F, GAF-S, PSP, PANSS, subscale PANSS, Negative Scale, Subscale PANSS Positive Scale, Subscale PANSS General Psychopathology Scale, HAM-D6, YMRS, CSQ, IMRS-P, IMRS-S, AHS, randomizing, sex, age, diagnosis, and CMHC. The automatic procedure in SPSS to produce missing imputed data values was used with 100 imputation estimates. Outliers were identified through Cooks’ D Diagram, and all outliers were included in all analyses. Residuals were tested for normality, linearity, and homoscedasticity, including using Spearman’s rank-order correlation. Also, complete case analysis was conducted where it is assumed that the missing data are missing completely at random and excluded in the analysis. In complete case analysis it is presumed to describe data where the observed complete cases are representative of the sample [31].

The per-protocol analysis was performed to explore whether attendance at the IMR groups influenced the results. One-way ANOVA was conducted to evaluate attendance at the IMR groups as a continuous variable and to treat attendance as a dichotomous variable (0–10 sessions vs. > 10 sessions); the latter measure was used for non-exposure vs. exposure. Subgroup analyses were conducted to evaluate whether the diagnosis or sex interacted with treatment for the secondary outcomes.

In addition, a post hoc analysis was performed to determine whether the subscale measurements of symptoms were influenced by IMR attendance as shown in other trials using one-way ANOVA [26]. The other post hoc analysis analyzed the IMRS as a three-factor solution. Also, we examined within-group changes at long-term follow-up by linear mixed model, with post-intervention and follow-up as repeated measurements, baseline as a fixed effect covariate, and an unstructured covariance matrix only focusing on time effect within groups.

Poisson regression analysis was used for the count outcomes (i.e., hospital admission, emergency room visits, and outpatient treatment). Descriptive analyses were used to explore whether the data were zero-inflated, and a negative binominal model was conducted when the data were zero-inflated. IBM SPSS Statistics (version 19) for Windows was used for the statistical analysis.

## Results

**Fig. 2:**
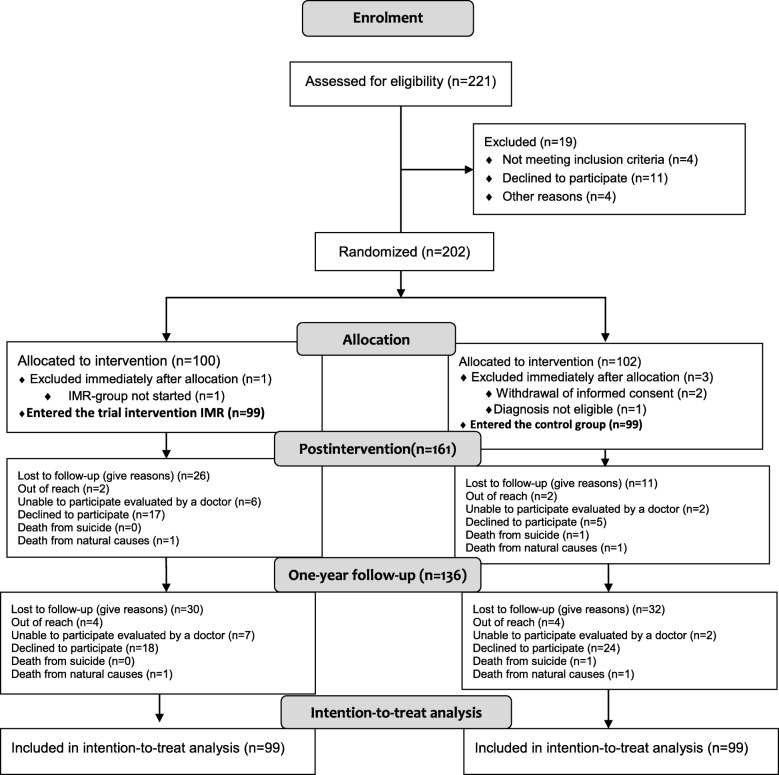
Flow Diagram for the Danish Illness Management and Recovery trial

Figure 2 shows the trial flowchart. A total of 198 patients completed the baseline interview, and 69% of the participants were available at the one-year follow-up. In total, 31 participants from the IMR group and 33 from the control group did not participate in the one-year follow-up. No differences were found in the demographic and clinical characteristics between the IMR and TAU groups at the one-year follow-up and Table 2 presents the baseline demographics on the participants.

**Table 2:** Demographic and clinical characteristics of the patients at the baseline

	Baseline			
	^b^IMR (*N* = 99)		TAU^c^ (*N* = 99)	
Variable	*N*	%	*N*	%
Site				
CMHC^a^ Ballerup	29	29.3	25	25.3
CMHC Gladsaxe	30	30.3	33	33.3
CMHC Frederiksberg	40	40.4	41	41.4
Sex				
Female	45	45.5	44	44.4
Age				
Age	41		45	
±SD	±11.0		±11.5	
Age range	20–68		22–77	
Housing				
Rented housing	75	75.8	65	65.7
Cooperative dwelling	14	14.1	18	18.2
Owner-occupied housing	8	8.1	10	10.1
Homeless	0		0	
Employment status				
Employed	7	7.1	12	12.1
Student	5	5.1	0	0
Unemployed or retired	84	84.8	81	81.8
Education				
Public school	26	26.3	26	26.3
High school	17	17.2	17	17.2
Vocational training	18	18.2	18	18.2
University	27	27.3	29	29.3
Living status				
Alone	70	70.7	69	69.7
Living with spouse and/or children	19	19.2	26	26.3
Diagnosis				
Schizophrenia	76	76.8	75	75.8
Bipolar disorder	23	23.2	24	24.2
Alcohol or drug abuse				
Alcohol or drug abuse	15	15.2	13	13.1
No abuse	80	80.8	80	80.8

^a^Community mental health center, ^b^Illness Management and Recovery, ^c^Treatment as usual

### Intent-to-treat analyses

Table 3 shows the results of the one-year follow-up, intent-to-treat analysis, and complete case analysis. In the GAF-F, a nonsignificant group indicated a difference of 0.8 in favor of the IMR group (95% confidence interval [CI]: − 4.7 to 3.0 points, t = − 0.42 *p* = 0.45). Table 3 summarized the analysis of the group means over time and revealed no significant differences between the IMR and control groups in clinical recovery symptoms (i.e., GAF-S, PSP, PANSS, HAM-D, and YMRS). Analyses of changes in personal recovery based on the MHRM, Hope, and Client’s Satisfaction Questionnaire also showed no differences between the IMR and control groups between either the baseline and one-year follow-up or the postintervention and one-year follow-up time points. As a post hoc analysis, we examined the time effect and while both groups improved on functioning and personal recovery, there was no differences on symptoms and illness management (data not shown).

**Table 3:** Recovery and illness self-management outcomes at the one-year follow-up

	Intent-to-treat analysis									Complete case analysis									
	One-year follow-up						Postintervention								One-year follow-up				
	IMR^a^			^b^TAU			IMR			TAU				IMR				TAU	
	*N*	Mean	Std. error	Mean	Std. error	*P*	*N*	Mean	SD	*N*	Mean	SD	*N*	Mean	SD	*N*	Mean	SD	*P*
Clinical recovery functioning																			
GAF-F^c^	99	50.6	1.38	49.8	1.40	0.67	62	45.4	12.1	59	43.5	11.8	66	50.4	13.4	62	50.2	11.8	0.63
Clinical recovery symptoms																			
GAF-S^d^	99	50.8	1.54	52.2	1.53	0.50	58	47.1	13.0	54	48.5	13.1	65	51.1	13.5	59	52.7	11.4	0.64
PSP^e^	99	52.1	1.55	53.1	1.57	0.63	59	52.6	13.4	59	49.6	14.1	64	52.3	14.2	63	53.5	13.1	0.25
PANSS^f^	99	56.5	1.83	52.8	1.83	0.15	51	57.5	15.4	52	60.4	18.9	55	58.7	18.1	57	57.6	20.7	0.51
Hamilton^g^	99	6.57	0.44	6.54	0.43	0.96	52	5.81	3.78	53	5.48	4.31	57	6.67	3.69	58	6.45	3.44	0.60
Young’s Mania^h^	99	8.56	0.75	8.0	0.74	0.47	52	6.77	5.92	53	7.49	6.20	57	8.02	5.34	58	7.43	5.60	0.40
	Intent-to-treat analysis									Complete case analysis									
Personal recovery																			
Mental Health Recovery Scale^i^	99	74.5	2.0	75.2	2.2	0.81	59	69.5	12.2	57	69.2	18.0	49	76.9	16.4	44	78.6	17.3	0.47
Adult State of Hope Scale	99	34.1	1.1	34.9	1.1	0.62	68	32.6	8.3	71	31.8	10.0	52	35.5	7.67	50	34.9	8.64	0.86
Patient satisfaction	99	24.4	0.52	25.3	1.0	0.84	61	24.6	5.1	58	24.8	4.1	55	25.0	4.76	46	25.8	4.0	0.28
Illness self-management																			
IMRS-Patient^j^	99	53.4	1.14	54.5	1.10	0.96	46	54.7	7.59	55	52.8	8.1	48	54.0	6.63	44	55.7	7.75	0.16
IMRS-Staff^k^	99	53.7	1.1	54.1	1.0	0.46	54	55.3	7.0	62	53.5	8.6	41	55.2	5.0	39	55.5	8.21	0.37

^a^Illness Management and Recovery;^b^Treatment as usual

^c^(GAF-F) Global Assessment of Functioning, Possible total scores range from 1 to 100 higher scores indicating higher level of functioning

^d^(GAF-S) Global Assessment of Symptoms, Possible total scores range from 1 to 100 higher scores indicating lower symptomology

^e^(PSP) Personal and Social Performance, Possible total scores range from 1 to 100 higher scores indicating better personal and social functioning

^f^(PANSS) Positive and Negative Syndrome Scale, Possible total scores range from 16 to 112

^g^(Hamilton) Hamilton Rating Scale for Depression, 6 items, ͬPossible total scores range from 6 to 48 higher scores indicating more severe symptomology

^h^(Young’s Mania) Young Mania Rating Scale, Possible total scores range from 0 to 60 higher scores indicating more severe symptomology

^i^Mental Health Recovery Scale. Possible total scores range from 30 to 150 with higher scores indicating better recovery

Adult State of Hope Scale. Possible total scores range from 8 to 48, where a higher number indicates greater hope

^j^Illness Management and Recovery Scale Patient version, ^k^Illness Management and Recovery Scale Staff version. Possible total scores range from 15 to 125 with higher scores indicating better illness management and recovery

Patient satisfaction. Possible total scores range from

n

### Complete case analyses

Similar results were found between the complete case analyses and the intent-to-treat analyses for the GAF-F, PSP, and PANSS. Furthermore, no between-group differences were observed in any of the other assessments, including the GAF-S, HAM-D, YMRS, Adult State of Hope, MHRM, Client’s Satisfaction Questionnaire, or the two versions of the IMRS.

### Service utilization

No significant differences were found in the number of hospital admissions (mean difference 0.49, 95% CI: 0.67–1.48, *p* = 1.0), days of hospital admission (mean difference 19.4 95% CI: -0.76-0.5, *p* = 0.5), or number of emergency room visits (mean difference 0.63, 95% CI: 0.81–1.54, *p* = 0.51) between the IMR and control groups.

### Fidelity

The IMR Fidelity mean scores across the three participating CMHCs assessed half-way at 4 months were CMHC 1: 4.3 (SD 0.9), CMHC 2: 4.1 (SD 0,1) and CMHC 3: 4.1 (SD 0.3) indicating good fidelity. The mean scores at the end assessment were CMHC 1: 4.2 (SD 0.7), CMHC 2: 4.0 (SD 0.8), and CMHC 3: 4.2 (SD 0.9) indicating high fidelity. For further information see Dalum et al. (2018) [17].

### Subgroup and subscale analyses

No association was found between a higher number of sessions attended and the GAF-F score at the one-year follow-up (see Table 4). The subgroup analyses showed no differences in the effect of IMR according to the participant diagnosis or sex on the outcomes of the GAF-F, PSP, or PANSS (see Table 4). We also performed post hoc analyses to investigate whether specific symptoms were influenced by IMR, as shown in other trials. When performing complete case analyses using univariate ANOVA with postintervention as a covariate, no significant difference was found between the intervention and control groups. A *p*-value of 0.05 was found for the PANSS positive symptoms favoring the TAU group; however, when considering the multiple statistical tests conducted, this result cannot be considered significant. We planned to analyze the IMRS as the total scale score (see Table 3), but post hoc we also analyzed the IMRS as three-factor solution subscales (the Recovery, Management, and Biology scales) [26]; however, the results were not significant (data not shown).

**Table 4:** Per-protocol and post-hoc subgroup analysis at one-year-follow-up

Per-protocol sub-group analysis								
Attendance in IMR		0–10 sessions (*N* = 20)			10+ sessions (*N* = 48)			
		Mean	SD	N	Mean	SD	N	P-value
GAF-F		47.3	13.7	19	51.3	12.8	47	0.26
PSP		48.5	12.5	19	53.4	14.4	45	0.20
PANSS		61.8	19.2	12	57.3	17.4	43	0.44
IMRS-Staff		54.0	5.1	9	54.9	6.9	32	0.73
Per-protocol sub-group analysis		IMR°			TAU•			
		Mean	SD	N	Mean	SD	N	P-value
Diagnosis Schizophrenia								
	GAF-F	47.4	12.2	49	48.6	11.8	52	0.60
	PSP	49.4	13.2	48	52.1	13.0	53	0.30
	PANSS	61.7	17.5	43	60.5	20.7	47	0.77
	IMRS-Staff	53.9	6.3	31	53.1	7.3	31	0.64
Bipolar Disorder								
	ͧGAF-F	58.1	12.7	17	57.7	8.87	10	0.94
	ͩPSP	59.8	13.4	16	60.8	9.83	10	0.83
	ͪPANSS	45.9	12.8	12	46.5	10.8	10	0.91
	ͮIMRS-Staff	57.1	6.9	10	62.6	3.29	8	0.06
Sex								
Male	GAF-F	47.4	11.9	39	50.9	12.4	42	0.21
	PSP	49.3	12.4	39	54.1	12.9	43	0.09
	PANSS	63.1	17.8	33	58.9	21.2	38	0.38
	IMRS-Staff	53.0	6.06	24	52.8	7.73	25	0.94
Female								
	GAF-F	54.0	14.0	27	48.5	10.7	20	0.15
	PSP	56.1	15.3	25	52.1	13.1	20	0.36
	PANSS	51.0	15.2	22	56.3	17.5	19	0.31
	IMRS-Staff	57.1	6.52	17	59.1	5.91	14	0.39
Post Hoc subgroup analysis		IMR			TAU			
		Mean	SD	N	Mean	SD	N	P-value
PSP	Aggressive behavior	0.58	0.75	62	0.50	0.68	60	0.49
	Activities	2.74	0.92	62	2.67	0.81	60	0.24
	Relations	2.00	1.24	62	1.97	1.1	60	0.68
	Self-care	1.13	0.97	62	1.25	1.0	60	0.46
PANSS	Positive	13.3	5.31	52	12.3	5.0	53	0.05
	Negative	14.4	6.3	51	14.0	5.87	52	0.63
	Psychopathology	31.2	8.75	52	31.2	14.7	53	0.95

о Illness Management and Recovery, •Treatment as usual, ͧ(GAF-F) Global Assessment of Functioning, ͩ(PSP) Personal and Social Performance, ͪ(PANSS) Positive and Negative Syndrome Scale, ͮIllness Management and Recovery Scale Staff version

## Discussion

The aim of the present study was to examine the long-term outcomes of an IMR trial, because the hypothesis behind the IMR program is that clinical and personal recovery can occur over a considerable timeframe after attending the program (Fig. 1). Overall, the study revealed no difference between the IMR and TAU groups in clinical recovery, personal recovery, illness self-management, and service utilization at the one-year follow-up. This result was similar to the findings at the end of the intervention [17].

### Fidelity and exposure to IMR

As mentioned in the introduction, two important issues should be considered in addition to the RCT design when interpreting the results: the fidelity of the IMR program and the participant’s exposure to the IMR program. The fidelity assessments showed good implementation at the program level [32]. The high fidelity can be explained by that all IMR facilitators went through a specific course of education prior to the intervention, had supervision during the intervention and held planned meetings during the intervention with the research team, leaders of the CMHC and IMR-instructors to evaluate the implementation of the IMR-program. Among the participants randomized to the IMR group, 13% did not attend a single IMR session, and 46.5% attended more than 20 sessions, resulting in an average session number of 16.4 (SD ±13.3) [17]. We had hoped for a higher participation rate. This result is a limitation, although the analyses showed no differences in the outcomes at the one-year follow-up between the participants who attended 0–10 sessions and those who attended 10+ sessions.

### Trial results in the context of previous IMR trials

In terms of symptoms, the review by McGuire et al. (2014) [7] found two RCTs [8, 9] supporting the hypothesis that IMR reduced symptoms, which aligned with the conclusion of a later study using a modified version of the IMR program [12]. However, the findings of the present study align with those of the study by Salyers et al. (2014) [11]. Thus, the results appear to be contradictory. Currently, a Cochrane review of IMR is being conducted; a meta-analysis will be conducted as part of this review if the symptom data are adequate, which will provide a better understanding of the effect of IMR on symptoms [33]. Furthermore, after a Dutch feasibility study of the IMR program, the authors proceeded to a RCT; the results from this RCT will contribute to clarifying the effect of IMR on symptoms [34].

The present study found no effect of IMR on improved functioning, which was in accordance with the study by Salyers et al. (2014) [11] but differed from the study by Levitt et al. (2009) [8]. Altogether, the results raise questions regarding the impact of IMR on functioning as a long-term effect. Personal recovery, such as hope [28], and a person’s experience with recovery (i.e., pursuing personal goals or taking social initiatives) were measured in three other trials, none of which reported significant improvement for the participants in IMR [9–11]. Therefore, the results of the present trial are in accordance with these results and suggest that IMR does not seem to impact the participants’ personal recovery at the long-term follow-up.

According to the conceptual framework underlying the IMR program, illness self-management is central for the ability of individuals to achieve clinical and personal recovery from mental illness [1]. All five trials assessed illness self-management with the IMRS. In three of the trials, both the patient (IMR-patient) and staff (IMR-staff) versions were used [8–10]. In contrast to our study, these three studies reported significant improvements resulting from IMR. Because the other RCTs had methodological limitations regarding the effect of IMR on illness self-management, examining the effect of IMR in a meta-analysis to obtain an integrated view of the effect is important [33]. The review by McGuire et al. (2014) [7] found that independent-assessor rated outcomes were more likely to show significant changes, although the present trial did not support these results. In our study we found time effect in both groups on functioning and personal recovery, but not on symptoms and illness management, indicating that services and/or time were generally associated with some improvement but IMR was not associated with additional benefit. One possible explanation for our null findings may be that the IMR-program is not as effective as earlier reviewer concluded. A recent Cochrane review showed that psychoeducational programs affected the participants’ symptom severity but not their daily functioning [35]. IMR is more than a psychoeducational program. In psychoeducational programs strategies are considered to be important for shared decision-making; however, other behavioral-based illness management strategies are also included in IMR, such as relapse prevention training and training in coping skills [36]. The results on the Cochran review on psychoeducational programs together with the results from IMR trials indicate the necessity of considering the need for programs with other conceptual frameworks to support people with severe mental illnesses in achieving functional and personal recovery. The results of present trial indicate that the IMR-program’s theoretical foundation, which is listed in Fig. 1 may be flawed.

### Strengths and limitations

The present RCT has several strengths. The trial was conducted according to the best methods available to reduce the risk of systematic, random, and design errors. The trial was conducted with adequate generation of the allocation sequence, adequate allocation concealment, adequate blinding wherever possible, adequate reporting of all relevant outcomes, and an intent-to-treat-analysis [37–40]. Moreover, sample size calculations were conducted prior to participant recruitment.

However, the trial also has some limitations. Although all centers reported high fidelity, reflecting good implementation of IMR in the CMHCs [32], none scored high on the fidelity item pertaining to the involvement of participants’ families or significant others [32]. A limitation in the fidelity assessment was that the IMR treatment integrity scale (IT-IS) to measure the therapeutic skills of the IMR-instructors or assessment to measure the implementation of IMR at organization level by the General organizational index (GOI-scale) was not applied in this trial. As previously mentioned, another limitation was that exposure to IMR was lower than we had hoped. However, no difference between the non-exposed participants (attending 0–10 sessions) and the exposed participants (attending 10+ sessions) was found at the one-year follow-up, which was in accordance with the fact that we also found no postintervention differences between the two groups. Postintervention, we performed post hoc analyses to address the exposure issue; however, none of our findings indicated that this problem had affected our results [17]. The exposure to IMR in the present study may better reflect a “real-world setting” than high IMR exposure. We can expect that some participants may not want to continue after a few IMR sessions and that some may not be able to participate in all sessions over a nine-month period. Furthermore, the waiting time from randomization to the first IMR group session was, on average, 87 days, which might have affected the results; indeed, providing psychosocial treatment when an individual is interested in and motivated to participate in such treatment is important. However, analyses to determine whether this wait affected the results did not indicate any effect [17]. In the present RCT, some of the IMR instructors were case managers for patients in the control group, and therefore, a spill-over effect in the control group was possible. However, the IMR instructors were educated to use only the IMR material for the IMR group participants. Furthermore, they were told to consult a well-experienced psychiatrist in only performing TAU, in case they were unsure whether specific elements could be regarded as part of TAU. A critical limitation is the high number of incomplete observed data due to a high lost to follow-up rate. Furthermore, a limitation is that nearly one-third of the participants did not complete the self-report instruments a long-term follow-up. To address these missing data, a mixed model with multiple imputations was generated to estimate and improve the statistical test values. Furthermore, the analyses were conducted according to the intent-to-treat principle, with the two-tailed level of significance for all statistical tests set at 0.05. Because all outcomes were exploratory, the risk of type 1 error was considerable. However, because none of the outcomes were statistically significant, the risk of type 1 error was ruled out. The participants who did not attend at the one-year follow-up may have recovered; however, these participants had significantly more hospitalizations, longer hospital admissions, and low attendance to the IMR program (see Table 4. When we conducted the present trial, we might have been too optimistic, because the trial was based on the number of participants needed to assess a 6-point difference on the GAF-F scale postintervention. Therefore, our sample size may be too small to state whether an observed difference of 0.8 is in fact a true difference at the one-year follow-up, but this finding most likely does not have clinical relevance.

## Conclusion

The present study showed no significant effect of IMR on clinical and personal recovery at the one-year follow-up. Together with previous RCTs with long-term follow-up, the findings raise questions about the effect of IMR on symptom severity. Altogether, the results of existing RCTs that have investigated IMR raise questions regarding the impact of IMR on functioning and do not suggest any effects on personal recovery. One possible explanation for our null findings is that the IMR-program is not as effective as earlier reviewer concluded. Hopefully, ongoing research on IMR [33, 41] will provide a more solid answer on this matter. However, the results suggest the need to consider the development of interventions based on other conceptual frameworks to help people with severe mental illnesses gain a better level of functioning and achieve personal recovery.

## Abbreviations

GAF-F: Global Assessment of Functioning–Functioning scale
GAF-S: Global Assessment of Symptom–Symptom scale
HAM: Hamilton Scale of Depression six items
Hope: Snider’ Adult Hope scale
IMR: The Illness Management and Recovery Program
IMRS: The Illness Management and Recovery Scale
MHRM: Mental Health Recovery Measure
NS: Not Significant
PSP: Personal and Social Performance scale
RCT: Randomized Clinical Trial
TAU: Treatment as Usual
YMRS: Young’s Mania Rating Scale

## References

[1] Mueser KT (2006). The illness management and recovery program: rationale, development, and preliminary findings. Schizophr Bull.

[2] Anthony W (1993). Recovery from mental illness: the guiding vision of the mental health service system in the 1990s. Psychiatr Rehabil J.

[3] Mueser KT, McGurk SR (2004). Schizophrenia. Lancet.

[4] Mueser KT (2013). Psychosocial treatments for schizophrenia. Annu Rev Clin Psychol.

[5] Andreasen NC (2005). Remission in schizophrenia: proposed criteria and rationale for consensus. Am J Psychiatry.

[6] Gingerich S. M.K.T., IMR Illness Management and Recovery Personalized Skills and Strategies for Those with Mental illness Implementation Guide. THIRD ed. Dartmouth PRC: Hazelden; 2011.

[7] McGuire AB (2014). Illness management and recovery: a review of the literature. Psychiatr Serv.

[8] Levitt AJ (2009). Randomized controlled trial of illness management and recovery in multiple-unit supportive housing. Psychiatr Serv.

[9] Fardig R (2011). A randomized controlled trial of the illness management and recovery program for persons with schizophrenia. Psychiatr Serv.

[10] Salyers MP (2010). Integrating assertive community treatment and illness management and recovery for consumers with severe mental illness. Community Ment Health J.

[11] Salyers MP (2014). A randomized controlled trial of illness management and recovery with an active control group. Psychiatr Serv.

[12] Tan CHS (2017). Illness management and recovery program for mental health problems: reducing symptoms and increasing social functioning. J Clin Nurs.

[13] Hasson-Ohayon I, Roe D, Kravetz S (2007). A randomized controlled trial of the effectiveness of the illness management and recovery program. Psychiatr Serv.

[14] Gingerich SMK (2003). Illness Management and Recovery.

[15] Ishak RB (2012). The effect of illness self-management and recovery program in reducing symptoms and increasing social functioning of the people with mental illness in the community: a preliminary study. Ann Acad Med Singap.

[16] Dalum HS (2011). Illness management and recovery (IMR) in Danish community mental health centres. Trials.

[17] Dalum HS (2018). Illness management and recovery: clinical outcomes of a randomized clinical trial in community mental health centers. PLoS One.

[18] Surtees PG, Kendell RE (1979). The hierarchy model of psychiatric symptomatology: an investigation based on present state examination ratings. Br J Psychiatry.

[19] Pedersen G, Hagtvet KA, Karterud S (2007). Generalizability studies of the global assessment of functioning-Split version. Compr Psychiatry.

[20] Nasrallah H, Morosini P, Gagnon DD (2008). Reliability, validity and ability to detect change of the personal and social performance scale in patients with stable schizophrenia. Psychiatry Res.

[21] Kay SR, Fiszbein A, Opler LA (1987). The positive and negative syndrome scale (PANSS) for schizophrenia. Schizophr Bull.

[22] Nicotra E (2015). On the use of the positive and negative syndrome scale in randomized clinical trials. Schizophr Res.

[23] Ruhe HG (2005). Clinical use of the Hamilton depression rating scale: is increased efficiency possible? A post hoc comparison of Hamilton depression rating scale, Maier and Bech subscales, clinical global impression, and symptom Checklist-90 scores. Compr Psychiatry.

[24] Young RC (1978). A rating scale for mania: reliability, validity and sensitivity. Br J Psychiatry.

[25] Lukasiewicz M (2013). Young mania rating scale: how to interpret the numbers? Determination of a severity threshold and of the minimal clinically significant difference in the EMBLEM cohort. Int J Methods Psychiatr Res.

[26] McGuire AB (2014). Rasch analysis of the illness management and recovery scale-clinician version. J Eval Clin Pract.

[27] Armstrong NP (2014). Validating a brief version of the mental health recovery measure for individuals with schizophrenia. Psychiatr Serv.

[28] Snyder CR (1991). The will and the ways: development and validation of an individual-differences measure of hope. J Pers Soc Psychol.

[29] Larsen DL (1979). Assessment of client/patient satisfaction: development of a general scale. Eval Program Plann.

[30] Søgaard HJ (1991). Psykiatrisk patientsregistrering Det psykiatriske centralregister i Danmark. En analyse af kritikken for og imod. Nordisk Psykiatrisk Tidsskrift.

[31] Pigott TD (2001). Missing predictors in models of effect size. Eval Health Prof.

[32] Dalum HS, et al. Participants' and staffs' evaluation of the illness management and recovery program: a randomized clinical trial. J Ment Health. 2016:1–8. 10.1080/09638237.2016.1244716 27841057

[33] Korsbek L, et al. Illness management and recovery programme for people with severe mental illness. Cochrane Database Syst Rev. 2014. 10.1002/14651858.CD011071.

[34] Roosenschoon BJ (2016). Illness Management & Recovery (IMR) in the Netherlands; a naturalistic pilot study to explore the feasibility of a randomized controlled trial. BMC Psychiatry.

[35] Zhao, S., et al., Psychoeducation (brief) for people with serious mental illness*.* Cochrane Database Syst Rev, 2015(4): p. CD010823. 10.1002/14651858.CD010823.pub2 PMC11053383 25854522

[36] Mueser KT (2002). Illness management and recovery: a review of the research. Psychiatr Serv.

[37] Higgens, J.G.S.e., The Cochrane Collaboration. Vol. version 5.1.0 [updated March 2011]. 2011: Cochrane handbook for systematic reviews of interventions.

[38] Lundh A, Sismondo S, Lexchin J (2012). Industry sponsorship and research outcome. Cochrane Database Syst Rev.

[39] Savovic J (2012). Influence of reported study design characteristics on intervention effect estimates from randomised controlled trials: combined analysis of meta-epidemiological studies. Health Technol Assess.

[40] Wood L (2008). Empirical evidence of bias in treatment effect estimates in controlled trials with different interventions and outcomes: meta-epidemiological study. BMJ.

[41] Roosenschoon BJ (2016). Effectiveness of illness management and recovery (IMR) in the Netherlands: a randomised clinical trial. BMC Psychiatry.

